# Interleukin-8 in Hyperlipidemia and Coronary Heart Disease in Thai Patients Taking Statin Cholesterol-Lowering Medication While Undergoing Coronary Artery Bypass Grafting Treatment

**DOI:** 10.1155/2020/5843958

**Published:** 2020-06-17

**Authors:** Wilanee Dechkhajorn, Yaowapa Maneerat, Kriengchai Prasongsukarn, Panan Kanchanaphum, Ratchanok Kumsiri

**Affiliations:** ^1^Department of Tropical Pathology, Faculty of Tropical Medicine, Mahidol University, Bangkok 10400, Thailand; ^2^Phramongkutklao Hospital, Bangkok 10400, Thailand; ^3^Department of Medical Science, Faculty of Science, Rangsit University, Pathumthani 12000, Thailand

## Abstract

The role of interleukin-8 (IL-8), a pivotal chemokine in atherogenesis and coronary heart disease (CHD) development, is diverse and remains unclear. This cross-sectional study investigates the association of the IL-8 expression in hyperlipidemia (H) and CHD patients who have been treated with statin cholesterol-lowering drugs while undergoing coronary artery bypass grafting treatment. Fifty-five Thai volunteers including 13 normal (N), 24 H, and 18 CHD patients were enrolled for the investigation. All the CHD patients had been treated continuously with statin cholesterol-lowering medications since the disease was diagnosed and were undergoing coronary bypass grafting approximately one month later. Therefore, the CHD group was representative of a pathogenesis improvement in CHD. The *IL8* mRNA expression was determined by real-time quantitative PCR in the peripheral blood mononuclear cells from heparinized blood. The plasma IL-8 levels were assessed by enzyme-linked immunosorbent assay. The result shows that the *IL8* mRNA expression in the H group tended to increase; however, in the CHD group, there was a significant decrease (*p*=0.0111) compared to the N group. The *IL8* mRNA expression and the plasma levels in the CHD group were significantly lower than those in the H group (*p* < 0.05). A significant negative correlation between the *IL8* mRNA (*r* = −0.499) or plasma IL-8 (*r* = −0.3875) expression and CHD progression was observed (*p* < 0.05). In conclusion, the transcriptomic and the phenotypic IL-8 expression decreased significantly in the Thai CHD patients who had continuously received statin-group medications compared to the H and N group participants. Therefore, IL-8 should serve as a feasible marker and could be used to evaluate the therapeutic effects of statins and illustrate the pathology of CHD treatment.

## 1. Introduction

Coronary heart disease (CHD) is one of the most significant vascular disorders in the world that is caused primarily by atherosclerosis complications. Major atherosclerosis disease mechanisms have been correlated to a high level of cholesterol, genetics, and the environment; however, the pathogenesis is still not understood entirely. Without treatment, atherosclerosis enhances occlusion of the arteries leading to ischemic heart disease and sudden myocardial infarction (MI) [1].

Extensive studies have investigated the role of inflammation in atherogenesis and coronary artery disease. The interplay of various inflammatory mediators, complements, and cytokines is involved in the pathogenesis of various stages of atherosclerosis from its initiation to progression, resulting in plaque formation and ruptures [2, 3]. These inflammatory molecules include interferon (IFN), colony-stimulating factors, interleukin (IL), chemokines, transforming growth factors (TGFs), and tumor necrosis factors (TNFs) (reviewed in [2, 4]). The proinflammatory cytokine IFN-*γ* can induce foam cell formation by increasing cholesterol retention through attenuating the expression of the ATP-binding cassette, subfamily A, as well as inducing the expression of acyl-CoA acyltransferase 1 that is involved in cholesterol esterification [4]. IL-1*α*, IL-1*β*, and IL-18 are proinflammatory molecules involved in atherosclerosis disease progression [5], while the anti-inflammatory cytokines TGF-*β*, IL-33, IL-10, and IL-13 function in the reduction of atherogenesis in cardiovascular disorders [4, 6].

IL-8 is a proinflammatory cytokine or chemokine (CXCL8) produced by various cell types including endothelial cells, peripheral blood monocytes, and vascular smooth muscle cells. IL-8 is encoded by *IL8* or *CXCL8* [7]. Prior research on patients and cell culture models [8, 9] has shown that IL-8 is involved in the pathogenesis of cardiovascular disorders including CHD [10], MI [11], strokes, [12], and other diseases such as ankylosing spondylitis [13], emphysema [14], periodontitis [15], and systemic lupus erythematosus [16]. In CVD, IL-8 participates in all stages of atherosclerosis and the development of CHD [17–20].

Although numerous studies have reported the atherogenic effect and the association of increased IL-8 expression with the development of cardiovascular disorders and CHD, the pivotal roles of IL-8 are still controversial. The anti-atherogenic effect of IL-8 on myocardial tissue repair was documented in MI [21–23], while IL-8 serum levels were correlated with a decrease in the occurrence of MI among women [11]. Consistent with this, prior research using monkeys as models showed that atherosclerotic plaques in carotid bifurcations were mildly inversely correlated with plasma IL-8 levels [24].

In this cross-sectional study, we investigated the association of *IL8* mRNA expression and the plasma IL-8 levels among healthy controls (N), hyperlipidemia patients (H), and CHD patients taking statin cholesterol-lowering medications while undergoing coronary bypass grafting in the CHD pathogenesis.

## 2. Materials and Methods

### 2.1. Materials

D-PBS (Wisent Inc., Quebec, Canada), TRIzol® Reagent (Invitrogen™, Carlsbad, CA, USA), IsoPrep (Robbins Scientific Corporation, Sunnyvale, CA, USA), and the RNeasy total RNA kit (Qiagen, Hilden, Germany) for RNA preparation were employed in this research. Real-time quantitative- (qRT-) PCR utilized primers that were designed according to GenBank sequences and based on previous studies [25, 26]. The primers were synthesized by Pacific Science Co., Ltd., Bangkok, Thailand. The human ELISA kit was purchased from BioLegend (San Diego, CA, USA). The other reagents used are described below.

### 2.2. Study Design, Study Population, and Ethical Considerations

**Figure 1:**
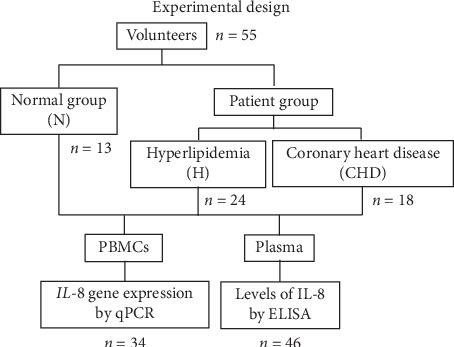
Experimental design.

The design of this study is summarized in Figure 1. This study comprised 55 male Thai citizens including 13 healthy volunteers (N group), 24 patients diagnosed with hyperlipidemia (H group), and 18 patients with coronary heart disease (CHD group). The patients were examined and treated by a physician from Phramongkutklao Hospital. They were classified based on their clinical manifestations according to the American College of Cardiology/American Heart Association criteria (2013) [27]. The H group had high cholesterol levels (total cholesterol (TC), LDL, and high-density lipoprotein (HDL)), but it showed no evidence of vital organ dysfunction. Before inclusion, it was confirmed that the participants from the N and H groups had not received any cholesterol or blood pressure-lowering medication. The CHD patients were about to undergo coronary bypass grafting under the supervision of KP. All the CHD patients had been treated continuously with statin drugs since they were diagnosed and after surgery: 8 of 18 patients received 20 mg/day of rosuvastatin and 10 of 18 patients received 40 mg/day of atorvastatin. The N and H patient groups did not have any infections or underlying diseases. The N group had no cardiovascular risk factors.

This study was approved by the RSU ethical review board (RSUERB2018-001), the Faculty of Tropical Medicine, Mahidol University (MUTM 2012-031-01), and Phramongkutklao Hospital (Q004q/55_Exp). Before enrollment, all the participants were informed of the aims and benefits of the study before completing an informed consent form.

### 2.3. Blood Sample Collection

Five milliliter of heparinized blood was collected from each participant of the three groups. Two milliliter of plasma was stored at –70°C for the detection of IL-8 by ELISA. The packed blood cells were resuspended in D-PBS and used to isolate the peripheral mononuclear cells (PBMCs). These were separated by Isoprep gradient centrifugation (Robbins Scientific Corporation) as described previously [28]. Approximately 2 million PBMCs in TRIzol (Invitrogen™) were kept at –70°C for the qRT-PCR.

### 2.4. Lipid Test

Lipid profiles including TC, triglyceride (TG), LDL cholesterol (LDL-c), and HDL cholesterol were determined by a biochemistry analyzer (Architect CI 16200, Abbott Laboratories, Abbott Park, IL, USA) at the clinical laboratory center of Phramongkutklao Hospital and/or by the Bangkok RIA Laboratory.

### 2.5. qRT-PCR Analysis of *IL8* mRNA Expression

The total RNA was isolated from the PBMCs using the RNeasy extraction kit (Qiagen) according to the manufacturer's instructions. The *IL8* mRNA was amplified by qPCR using the SuperScript™ III One-Step RT-PCR System with Platinum™ Taq DNA Polymerase according to the manufacturer's instructions (Invitrogen™, Carlsbad, CA, USA). Amplification was conducted in a Bio-Rad CFX Connect Real-Time System (Bio-Rad Laboratories, Inc., Hercules, CA, USA) using the following primers: cIL-8F (forward) 5′-GGCACAAACTTTCAGAGACAG-3′ and cIL-8R (reverse) 5′-ACACAGAGCTGCAGAAATCAGG-3′ [25]; glyceraldehyde 3-phosphate dehydrogenase (*GADPH*) was used as a housekeeping gene to normalize the relative expression of *IL8* with the primers: G6F (forward) 5′-ACAGAGTGAGCCCTTCTTCAA-3′ and G6R (reverse) 5′-GGAGGCTGCATCATCGTACT-3′ [26]. PCR conditions were 94°C for 2 min followed by 40 cycles of 95°C for 15 s, 60°C for 30 s, and 76°C for 60 s. Calculations of relative expression levels were performed using the 2-^ΔΔCt^ method [28]. The qRT-PCR reactions were performed in quadruplicate.

### 2.6. Determination of Plasma IL-8 Levels Using ELISA

Levels of plasma IL-8 were determined by Human IL-8 ELISA MAX™ Deluxe (BioLegend, San Diego, CA, USA) according to the manufacturer's recommendations. In brief, 96 wells were coated with a capture antibody and incubated overnight at 2–8°C. Plasma samples (100 *μ*L) from the three groups and prediluted standards were added and incubated for 2 h at room temperature. Then, the wells were washed to remove any unbound material, and 100 *μ*L of diluted detection antibody was added for 60 min at room temperature. After the unbound materials were removed, 100 *μ*L of avidin-horseradish peroxidase conjugate was added for 30 min at room temperature. The bound enzyme was detected by adding 100 *μ*L of substrate solution C to each well and the reactions were stopped by adding 100 *μ*L of stop solution. The optical density was determined at 450 nm using the Tecan Sunrise OEM Remote Microplate Absorbance Reader (Tecan, Grödig, Austria). All the samples were assayed in triplicate. The IL-8 levels were calculated from standard curves.

### 2.7. Statistical Analysis

Clinical data were presented as medians (upper and lower range limits). The *IL8* mRNA expression was represented as a fold change relative to the *GADPH* mRNA expression in the PBMCs. The IL-8 protein levels were expressed as a mean ± SEM. The significant difference between two or more groups was determined by the Mann–Whitney *U* test and the Kruskal–Wallis test, respectively. The correlations between CHD development/improvement and the *IL8* mRNA expression or the plasma IL-8 levels were determined by Spearman's rho correlation analysis. Significance was set at *p* < 0.05 at a 95% confidence interval. All statistical analyses were performed using SPSS software version 18 (SPSS, Chicago, IL, USA).

## 3. Results

### 3.1. Clinical Manifestations

The patients' characteristics, clinical manifestations, and the healthy controls were compared between groups and summarized, as shown in Table 1. There was no significant difference in age between the N and H groups (*p* > 0.05). The CHD patients were significantly older than the participants in the N and H groups. The levels of TC (*p* < 0.0001) and LDL (*p*=0.0002) were significantly higher in the H group than in the N group, and the levels of “bad” lipids including TC (*p*=0.0028), TG (*p*=0.0183), and LDL (*p*=0.045) were significantly higher in the H group than in the CHD group. The CHD patients had a lipid baseline that was not significantly different when compared to N group participants, except for TG (*p*=0.0387).

### 3.2. *IL8* mRNA Expression

**Figure 2:**
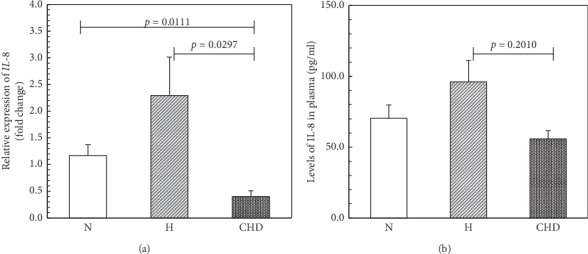
IL-8 expression and its association to clinical manifestation. (a) *IL8* mRNA expression (2.0-fold change) relative to *GADPH* mRNA in peripheral blood mononuclear cells obtained from normal controls N, hyperlipidemia H, and patients with acute coronary heart disease (CHD), as determined by qRT-PCR. Calculations of relative expression levels were performed using the 2-^ΔΔCt^ method [26]. qRT-PCR reactions were performed in quadruplicate. (b) Plasma levels (pg/ml) of IL-8 from N, H, and CHD groups using ELISA. The assay was performed in quadruplicate. Data are presented as mean ± SEM.

The *IL8* mRNA expression levels are represented as relative expression (fold change) (Figure 2(a)). The *IL8* mRNA expression in the CHD group was significantly lower than that in the N (*p*=0.0111) and H groups (*p*=0.0297). The *IL8* mRNA expression tended to be higher in the H group than in the N group (*p* > 0.05).

### 3.3. Levels of IL-8 in Plasma

The levels of plasma IL-8 in all the groups are shown in Figure 2(b). Similar to the *IL8* pattern expression, the level of IL-8 was higher in the H group compared to the N (*p* > 0.05) and CHD (*p*=0.0210) groups. Levels did not differ significantly between the N and the CHD groups (*p* > 0.05).

### 3.4. Correlation between the IL-8 Expression and CHD Improvement

There was a significant negative correlation between *IL8* mRNA (*r* = −0.499, *p*=0.0248) or the plasma IL-8 level (*r* = −3875, *p*=0.0195) and CHD improvement. The IL-8 and other parameters did not show significant correlation.

## 4. Discussion

This cross-sectional study used N, H, and CHD groups to illustrate atherogenesis development from healthy to atherosclerosis (moderate risk of CHD), consequent CHD, and the final improvement of plaques in coronary arteries. This is in accord with previous studies, which found that the reduction in the LDL-C level and the degree of anti-inflammation are inversely related to atherosclerotic progression [29, 30]. We chose only male volunteers to control the influence of the sex-hormone factor such as estrogen. Because estrogens are primary examples of female sex steroids, the epidemiological studies in animal models have shown that estrogen has protective effects in cardiovascular disease. Previous evidence has also suggested that estrogen protects women against CHD premenopause (reviewed in [31]).

Although research into the functions of IL-8 has proved controversial, most previous studies reporting the contribution of the IL-8 expression to the progression of cardiovascular disorders involved both acute and chronic vascular inflammation [17–20]. IL-8 is a cytokine that is released in high amounts from neutrophils that participate in all stages of atherosclerosis and the development of CHD [17–19]. IL-8 is mediated through various mechanisms that contribute to cardiovascular disorders [12–20, 32–36]. IL-8 from neutrophils was found in both the circulation and the intraplaque of carotid stenosis. This finding indicates that IL-8 is critical for plaque development and progression [37]. In atherogenesis, IL-8 is a chemoattractant [36] that promotes monocyte adherence to the vascular endothelium [33] and has mitogenic effects on vascular smooth muscle cells [36]. IL-8 controls the cholesterol efflux via upregulating miR-183 in macrophage-derived foam cells [33]. IL-8 is also involved in the migration of monocytes into the subendothelial space which is a crucial process in the early stages of atherosclerosis [32, 38]. IL-8 is mediated through the downregulation of the tissue inhibitor metalloproteinase-1 to contribute atherogenesis (reviewed in [26, 34]). Moreover, the polymorphism of IL-8-251A/T contributes to CHD susceptibility in Chinese populations [20, 21].

In acute inflammation, IL-8 functions in chemotaxis and induces migration of neutrophils and macrophages to the tunica intima of the subendothelial space, sites of injury, or endothelial fatty streaks [17]. Localization of monocyte chemoattractant protein-1 (MCP-1) and IL-8 is found in human atheroma. An earlier *in vitro* study proved that IL-8 was a powerful trigger for firm adhesion of monocytes to the vascular endothelium under flow conditions [33]. In addition, elevated baseline plasma levels of IL-8 have been associated with an increased risk of long-term all-cause mortality in patients with acute coronary syndrome. Furthermore, this association was independent of a variety of clinical, laboratory, and angiographic variables including contemporary biomarkers with established prognostic efficacy in acute coronary syndrome [10].

Statins are selective competitive inhibitors of 3-hydroxy-3-methyl-glutaryl-CoA reductase that interfere with mevalonate-derived compounds and lower cholesterol. Therefore, statins are used to treat hypercholesterolemia and are involved in the primary and secondary prevention of cardiovascular diseases. Rosuvastatin and atorvastatin are newly developed statins, and the former can lower LDL-C more effectively than the latter (reviewed in [39]). This study showed that CHD patients taking 20 mg of rosuvastatin or 40 mg of atorvastatin achieved the LDL-C goal (<100 mg/dl). These findings were in line with previous 30-day treatment studies [40, 41]. Also, recent research has revealed that the LDL-C goal was not time-dependent [42].

Several previous studies found that statin drugs have played a role in the anti-inflammation of cardiovascular disorders [39, 43, 44]. Atorvastatin was shown to attenuate neutrophil migration after two weeks of exposure to a high dose (80 mg) and to decrease the IL-8 expression in a dose-dependent manner [43]. In addition, statins inhibit leukocyte and endothelial cell interactions and reduce inflammatory cell numbers within atherosclerotic plaques (reviewed in [39]. Prior research has suggested that rosuvastatin suppresses the inflammatory response through a mechanism involving the inhibition of c-Jun N-terminal kinase and NF-*κ*B. Moreover, it has been found to reduce adhesion molecules and some cytokines including IL-8, IL-6, and MCP-1 [44]. Due to the ability of the statin group to reduce inflammatory cytokine production, statin therapy has been applied as an alternative in some infectious diseases to prevent overuse-related antibiotic resistance, e.g., in intestinal *Salmonella* infections [45], *Pseudomonas* pneumonia [46], and chronic obstructive pulmonary disease (COPD) [47].

The therapeutic effects of rosuvastatin and atorvastatin reduce bad lipids, decrease vascular inflammation, and result in the regression of coronary atherosclerotic plaques [48]. In this meta-analysis study, intravascular ultrasound imaging demonstrated that both drugs could significantly reduce atheroma volume, increase the lumen volume of the coronary artery, and regress the composition of atherosclerotic plaques. Regression and stabilization of plaque prevent rupture that leads to vascular occlusion.

In our cross-sectional study, we found that the *IL8* mRNA expression and the IL-8 plasma protein levels increased concordantly in the H group compared to healthy control. In contrast, we observed a significant decrease in the *IL8* mRNA expression (*p*=0.0126) and the corresponding protein levels (*p*=0.0247) in CHD patients compared with the H group participants. We assume that the decrease in the IL-8 expression in the CHD group was caused by the action of cholesterol-lowering medications, as previously described by patients taking rosuvastatin and atorvastatin [44]. These results concur with Yeh et al. who recently reported a lower CAD risk among all statin users that was not duration dependent [42].

A previous study has demonstrated that IL-18 was a potential predictive marker for atherosclerosis progression in SIV-infected rhesus monkeys (*Macaca mulatta*) on a high-fat/high-cholesterol diet [25]. Similarly, we suppose that the reduced IL-8 expression among the statin-group-treated patients for CHD might illustrate the chronic inflammation regression of coronary arteries and other vasculature. Even though the regression of atherosclerotic plaque was not ultrasonically tested, we assumed that after treatment with rosuvastatin and atorvastatin, the pathologic changes of plaque in the coronary arteries were signs of recovery. Prior research agrees with our hypothesis that statin treatment not only decreases LDL but also results in plaque improvement [48, 49].

This study has several limitations. First, the study did not include CHD patients who had never been treated with statins or other cholesterol-lowering drugs. We lack some information from the patients' characteristics such as the detail of coronary graft bypass or clinical history and the progress of disease. However, reliable literature has mostly reported an increase in the *IL8* mRNA expression and IL-8 levels in cardiovascular diseases and coronary artery syndrome. IL-8 is mediated through various mechanisms to contribute to cardiovascular disorders [12–20, 32–36]. Second, the size of the study population was small and not designed using a priori calculations of statistical power. Therefore, these findings need to be confirmed with larger sample sizes in further studies. Third, in this study, we did not determine the pathologic change in the atherosclerosis plaques in the CHD patient group. Therefore, we could not confirm the improvement of plaque in the vessels after treatment although previous studies suggest that the therapeutic effects of rosuvastatin and atorvastatin result in the regression of coronary atherosclerotic plaques [48]. Fourth, other pivotal cytokines, such as TNF-*α*, MCP-1, IL-1*β*, and IL-6, should be considered for future research [2].

## 5. Conclusion

This study found that both the transcriptomic and the phenotypic IL-8 expression increased in hyperlipidemia. In contrast, a significant decrease in the amount of IL-8 was observed in Thai CHD patients who took statin medication. Based on our findings and previous studies on the role of IL-8 in the pathogenesis of CVD, IL-8 may have the potential to be used as an alternative marker to evaluate the therapeutic effects of the statin drug group.

## Figures and Tables

**Table 1:** General description and clinical manifestations of the study population and the comparison between groups.

Variable	Normal	Hyperlipidemia	Coronary heart disease	*p* value		
	(N)	(H)	(CHD)			
	(*n* = 13)	(*n* = 24)	(*n* = 18)	N vs. H	H vs. CHD	N vs. CHD
Age (year)	45.5 (23–59)	41 (26–58)	65.5 (58–78)	0.5577	**<0.0001**	**<0.0001**

TC (mg/dL)	178.5 (150–199)	226.5 (200–344)	171 (115–259)	**<0.0001**	**0.0028**	0.6548

TG (mg/dL)	164.5 (76–242)	172 (70–280)	84 (65–169)	0.9157	**0.0183**	**0.0387**

HDL (mg/dL)	42.5 (26–60)	45 (30–80)	49 (39–75)	0.5276	0.2624	0.1576

LDL (mg/dL)	113 (63–124)	145 (70–190)	90 (44–174)	**0.0002**	**0.045**	0.6932

All normal controls and patients were male. N = normal controls; H and CHD = patients with hyperlipidemia and coronary heart disease, respectively. TC = total cholesterol; TG = triglyceride; HDL = high-density lipoprotein; LDL = low-density lipoprotein. Data are shown as median (ranges). The differences in each variable between two groups (N vs. H, H vs. CHD, and N vs. CHD) were determined using the Mann–Whitney *U* test. The *α* level was set at <0.05 at a 95% confidence interval.

## Data Availability

The data used to support the findings of this study are available from the corresponding author upon request.
